# Quantifying climate conditions for the formation of coals and evaporites

**DOI:** 10.1093/nsr/nwad051

**Published:** 2023-02-27

**Authors:** Xiujuan Bao, Yongyun Hu, Christopher R Scotese, Xiang Li, Jiaqi Guo, Jiawenjing Lan, Qifan Lin, Shuai Yuan, Mengyu Wei, Zhibo Li, Kai Man, Zihan Yin, Jing Han, Jian Zhang, Qiang Wei, Yonggang Liu, Jun Yang, Ji Nie

**Affiliations:** Laboratory for Climate and Ocean-Atmosphere Studies, Department of Atmospheric and Oceanic Sciences, School of Physics, Peking University, Beijing 100871, China; Laboratory for Climate and Ocean-Atmosphere Studies, Department of Atmospheric and Oceanic Sciences, School of Physics, Peking University, Beijing 100871, China; Department of Earth and Planetary Sciences, Northwestern University, Evanston, IL 60208, USA; Laboratory for Climate and Ocean-Atmosphere Studies, Department of Atmospheric and Oceanic Sciences, School of Physics, Peking University, Beijing 100871, China; Laboratory for Climate and Ocean-Atmosphere Studies, Department of Atmospheric and Oceanic Sciences, School of Physics, Peking University, Beijing 100871, China; Laboratory for Climate and Ocean-Atmosphere Studies, Department of Atmospheric and Oceanic Sciences, School of Physics, Peking University, Beijing 100871, China; Laboratory for Climate and Ocean-Atmosphere Studies, Department of Atmospheric and Oceanic Sciences, School of Physics, Peking University, Beijing 100871, China; Laboratory for Climate and Ocean-Atmosphere Studies, Department of Atmospheric and Oceanic Sciences, School of Physics, Peking University, Beijing 100871, China; Laboratory for Climate and Ocean-Atmosphere Studies, Department of Atmospheric and Oceanic Sciences, School of Physics, Peking University, Beijing 100871, China; Laboratory for Climate and Ocean-Atmosphere Studies, Department of Atmospheric and Oceanic Sciences, School of Physics, Peking University, Beijing 100871, China; Laboratory for Climate and Ocean-Atmosphere Studies, Department of Atmospheric and Oceanic Sciences, School of Physics, Peking University, Beijing 100871, China; Laboratory for Climate and Ocean-Atmosphere Studies, Department of Atmospheric and Oceanic Sciences, School of Physics, Peking University, Beijing 100871, China; Laboratory for Climate and Ocean-Atmosphere Studies, Department of Atmospheric and Oceanic Sciences, School of Physics, Peking University, Beijing 100871, China; Laboratory for Climate and Ocean-Atmosphere Studies, Department of Atmospheric and Oceanic Sciences, School of Physics, Peking University, Beijing 100871, China; Laboratory for Climate and Ocean-Atmosphere Studies, Department of Atmospheric and Oceanic Sciences, School of Physics, Peking University, Beijing 100871, China; Laboratory for Climate and Ocean-Atmosphere Studies, Department of Atmospheric and Oceanic Sciences, School of Physics, Peking University, Beijing 100871, China; Laboratory for Climate and Ocean-Atmosphere Studies, Department of Atmospheric and Oceanic Sciences, School of Physics, Peking University, Beijing 100871, China; Laboratory for Climate and Ocean-Atmosphere Studies, Department of Atmospheric and Oceanic Sciences, School of Physics, Peking University, Beijing 100871, China

**Keywords:** deep-time climate evolution, coals, evaporites, temperature, precipitation

## Abstract

Coals and evaporites are commonly used as qualitative indicators of wet and dry environments in deep-time climate studies, respectively. Here, we combine geological records with climate simulations to establish quantitative relationships of coals and evaporites with temperature and precipitation over the Phanerozoic. We show that coal records were associated with a median temperature of 25°C and precipitation of 1300 mm yr^−1^ before 250 Ma. Afterwards, coal records appeared with temperatures between 0°C and 21°C and precipitation of 900 mm yr^−1^. Evaporite records were associated with a median temperature of 27°C and precipitation of 800 mm yr^−1^. The most remarkable result is that net precipitation associated with coal and evaporite records remained constant across time. The results here have important implications for quantifying climate conditions for other lithologic indicators of climate and for predicting exogenetic ore deposits.

## Teaser

Quantitative relationships of coals and evaporites with temperature and precipitation can be established. Net precipitation associated with coals and evaporites remained constant across time.

## INTRODUCTION

It is well known that coals, as the lithified and compressed fossils of plant materials, are usually associated with warm and humid environments [1]. The formation of coals involves three stages: plants, peats, and lithified coals. As the precursors of peats and coals, plant productivity is closely tied to climate conditions, i.e. temperature and precipitation, while peat formation from plants is associated with biochemical processes that require a stable water environment to prevent desiccation and oxidation of accumulating plant materials, and lithification from peats to coals is mainly related to physical and geological process [1]. Thus, the relationship between coals and climate conditions more accurately means the relationship of plant productivity with surface temperature and precipitation. In contrast, evaporites (primarily halite and potassium chloride) are deposited in hot and arid regions [2]. For these reasons, the geological records of coals and evaporites have been qualitatively used as temperature and precipitation proxies in deep time climate studies [1–9]. However, how coals and evaporites are quantitatively associated to temperatures and precipitation in the deep time has not been established.

The purpose of the present paper is to quantify the relationships of coals and evaporites with surface temperature and precipitation during the Phanerozoic, by combining the geological records of coals and evaporites with the results of our climate simulations [10,11]. Geological records of coals range in age from the present-day back to the Early Devonian (410 million years ago (Ma)), and evaporites occurred throughout the Phanerozoic [6,12] (Supplementary Fig. 1). Simulated annual mean temperature (AMT) and annual mean precipitation (AMP) for 55 high-resolution time-slice simulations, representing changing paleogeographic conditions during the last 540 million years (Myr) (one every 10 Myr), are documented in Li *et al.* (2022) [10,11]. Simulated global AMTs are nearly the same as reconstructions [9], and simulated global AMP of the pre-industrial period (PI) is 1062 mm yr^−1^ that is close to present-day observations of 982 mm yr^−1^ (see Method) [13]. The corresponding global maps of net precipitation are shown in Supplementary Fig. 2. A detailed description of coal and evaporite records, as well as the simulation results, can be found in the Data and Methods section.

## LATITUDINAL DISTRIBUTION OF COALS AND EVAPORITES

**Figure 1.**
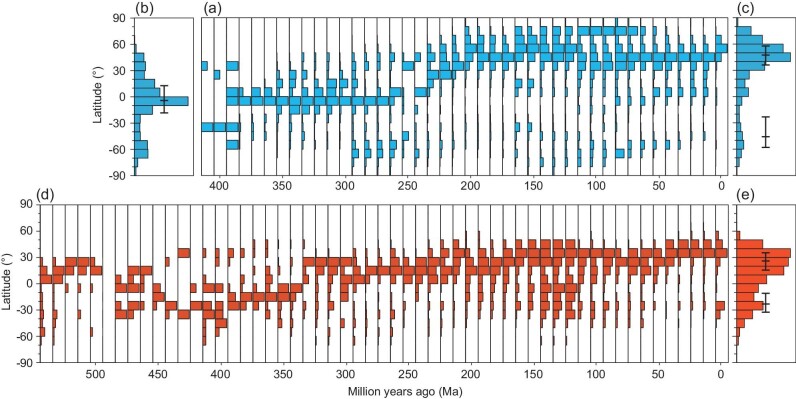
Latitudinal distributions of coals and evaporites during the Phanerozoic. (a) Latitudinal distributions of coals during the Phanerozoic, (b) histogram of coal occurrences from 410 Ma to 250 Ma, (c) histogram of coal occurrences between 240 Ma and present day, (d) latitudinal distributions of evaporites during the Phanerozoic, (e) a histogram of evaporites during the Phanerozoic. Note that the tallest bar for each time slice denotes the maximum number of coal or evaporite records. In plots (b), (c) and (e), the latitudes containing 25%, 50% and 75% of the coal and evaporite records are marked by the vertical black bars. In plots (c) and (e), the evaporite statistics have been calculated separately for the Northern and Southern Hemispheres.

Though plants had invaded the land by the late Ordovician–earliest Silurian (440 Ma), coal producing ecosystems did not become established until the Early Devonian (410 Ma) [14,15]. The latitudinal distribution of coals during the past 410 million years is shown in Fig. 1a. It is clear that there was an important change in the latitudinal range of coals after 250 Ma. Coals occurred predominantly in the equatorial region before 250 Ma. The median latitude of late Paleozoic coals was 4°S (Fig. 1b); 50% of coal records in the dataset occurred between 18°S and 13°N. During the Triassic, the location of coals rapidly shifted to the Northern-Hemisphere (NH) rainy zone at middle and high latitudes. The median latitude of NH coals was 48°N (Fig. 1b), and 50% of coal records were located between 36°N and 58°N. The median latitude of Southern-Hemisphere coal records is 46°S, and 50% of the coal records was located between 23°S and 58°S.

The change in the latitudinal distribution of coals can be attributed to three factors: 1) the replacement of pteridophytes by gymnosperms, 2) the evolution of white fungi (Agaricomycetes), and 3) the movement of landmasses into the NH. Pteridophytes, with low water transport capacity, were the dominant plants before 250 Ma [16,17], and they preferred to grow in the tropics (0°–15°N–S) where climate was warm and humid. Thus, coals were mainly formed by pteridophytes in tropical latitudes in the late Paleozoic (360 Ma–280 Ma). In the Mesozoic, gymnosperms became dominant. Internal water transport systems–secondary xylem had been developed in gymnosperms, making plants more adaptable to drier and cooler conditions than pteridophytes, so that coals expanded into the rainy temperate belts at higher latitudes (40°–80°N and S) [18]. Coincident with this evolutionary transition, continents gradually moved into the NH, providing a much larger habitat for coal-formation. Therefore, during the Mesozoic and Cenozoic more coals began to be deposited in the NH rainy zone (∼50°N).

White rot fungi, the only organism capable of substantial lignin decay, originated at ∼295 Ma based on Molecular clock analyses [19]. The sudden reduction in the number of tropical coals after the beginning of the Permian is thus likely to be due to the extinction of early coal-forming plants and the evolution of white rot fungi that caused the accelerated decay of lignin. It should also be noted that the drop in coal abundances in the early Triassic and the ensuing ‘coal gap’ can be directly attributed to the extinction event at the Permian–Triassic boundary (Supplementary Fig. 3).

Figure 1b and c are histograms that illustrate the latitudinal extent of coals during the late Paleozoic (410 Ma–250 Ma) and the Mesozoic and Cenozoic (240 Ma–0 Ma), respectively. The two plots clearly show the rapid shift of coals from the equatorial region to about 50°N. The angiosperm terrestrial revolution in the late Cretaceous and early Paleogene promoted a new level of terrestrial plant productivity [20–23]. Coal deposition exceeded the rate of lignin-degradation in the tropics and coals, once again, became more common.

Unlike the changing latitudinal range of coals, evaporites have always formed in the subtropical dry zones of the Northern and Southern hemispheres (Fig. 1d). After 340 Ma, there was a notable shift in the latitudinal occurrence of evaporites. Previous to the early Carboniferous, nearly all evaporites formed in the southern subtropics. In the late Paleozoic, the occurrence of evaporites shifted from the southern subtropics to the northern subtropics (Supplementary Fig. 4). This shift was due to the northward motion of the Pangea supercontinent and the increasing area of subtropical regions in the NH during the Mesozoic and Cenozoic (Fig. 1; Supplementary Fig. 1; Supplementary Fig. 4).

## RELATIONSHIPS OF COALS AND EVAPORITES WITH TEMPERATURES

**Figure 2.**
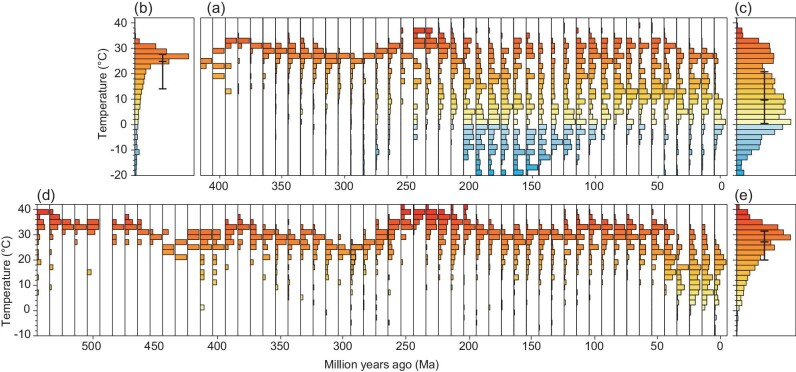
Distributions of coals and evaporites as a function of AMT during Phanerozoic. (a) Coals, (b) histogram of coals over 410 Ma–250 Ma, (c) histogram of coals over 240 Ma–present, (d) evaporites, (e) histogram of evaporites over 540 Ma–present. Colors indicate AMTs. The temperatures containing 25%, 50%, and 75% of the coal and evaporite records are marked by the vertical black bars in (b), (c) and (e).

Figure 2a shows the abundance of coals as a function of AMT during the past 410 Myr. Before 250 Ma, the median temperature associated with Paleozoic coals was 25°C, and 50% of coal records in the dataset were associated with temperatures between 14°C and 28°C (Fig. 2b). After 250 Ma, coal records were associated with a much broader range of surface temperatures (Fig. 2c). The median temperature was 10°C, and 50% of coal records appeared within a temperature range of 0°C and 21°C. The reason for this dramatic change in the range of surface temperatures is the northward latitudinal shift of coal localities shown in Fig. 1a. In the Paleozoic, coals were primarily located in hot, steamy tropical environments. In contrast, coals formed at much cooler and rainy latitudes (50°N–S) after 250 Ma.

Figure 2d shows the relationship of evaporites with AMTs during the Phanerozoic. The temperatures associated with evaporites were quite variable across time. These temperature variations reflect the changing global mean surface temperature (GMST) [9,22] (Supplementary Fig. 5). For example, GMSTs were lower during the late Carboniferous and early Permian icehouse (340 Ma–280 Ma) as well as during the late Cenozoic ice age (50 Ma–0 Ma), but were higher during the hothouse intervals of the Cambrian, Devonian, Triassic, and Cretaceous. The median temperature associated with evaporites was 27°C, and 50% of evaporite records appeared within a temperature range of 20°C–31°C (Fig. 2e). The evaporite-related temperatures represent temperatures that characterize the subtropical belt temperature during the Phanerozoic.

## RELATIONSHIPS OF COALS AND EVAPORITES WITH PRECIPITATION

**Figure 3.**
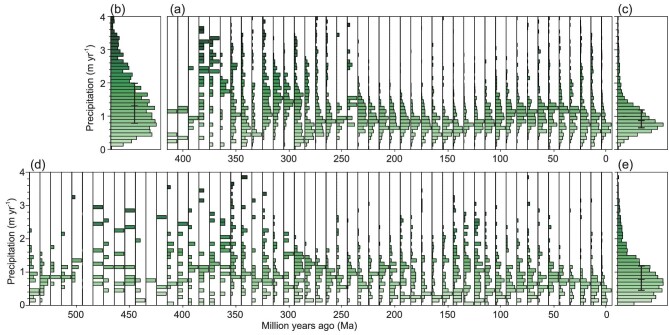
Distributions of coals and evaporites as a function of AMP. (a) Coals, (b) histogram of coals over 410 Ma–250 Ma, (c) histogram of coals over 240 Ma–present, (d) evaporites, and (e) histogram of evaporites over 540 Ma–present. The precipitation values containing 25%, 50% and 75% of the coal and evaporite records are marked by the vertical black bars in (b), (c) and (e).

Figure 3a shows the relationship between coals and AMP during the past 410 Myr. As in the case with temperature, there was an important turning point at 250 Ma. Prior to 250 Ma, coals were associated with a broad range of AMP. The median precipitation during the Paleozoic was 1300 mm yr^−1^, and 50% of coal records occurred within the precipitation range between 800 mm yr^−1^ and 2000 mm yr^−1^. The highest values represented precipitation associated with the intertropical convergence zone (Fig. 3b). In the Mesozoic and Cenozoic, coal-related precipitation values were ∼900 mm yr^−1^, and 50% of the coal records fell within a range of precipitation between 700 mm yr^−1^ and 1200 mm yr^−1^ (Fig. 3c).

Coal-related precipitation (Fig. 3a) appeared to be most variable during the Paleozoic (i.e. prior to 250 Ma). In contrast, there are only a few periods of heavy coal-related precipitation during the late Cretaceous and Cenozoic. Interestingly, during much of the Mesozoic (from the Triassic to the early Cretaceous) coals were characterized by low precipitation values.

The distribution pattern of precipitation for evaporites (Fig. 3d) was similar to coals and largely tracked GMST. That is to say, higher precipitation values were observed during cooler periods (e.g. late Paleozoic, late Jurassic–early Cretaceous) or at times when there was more land at higher latitudes (throughout the Paleozoic). Overall, for evaporites the median precipitation was 800 mm yr^−1^, and 50% of the evaporite records appeared within the precipitation range between 500 mm yr^−1^ and 1200 mm yr^−1^ (Fig. 3e).

## RELATIONSHIPS OF COALS AND EVAPORITES WITH NET PRECIPITATION

**Figure 4.**
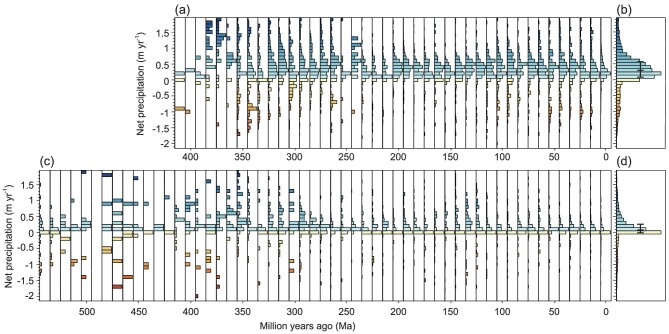
Distributions of coals and evaporites as a function of annual mean net precipitation. (a) Coals, (b) histogram of coals over 410 Ma–present, (c) evaporites, and (d) histogram of evaporites over 540 Ma–present. The net precipitation values containing 25%, 50% and 75% of the coal and evaporite records are marked by the vertical black bars in (b) and (d).

It is clear from the distribution patterns of temperature and precipitation described in the previous section that coals and evaporites tend to form in very different climatic environments. Coals appeared in ‘wetter’ environments, and evaporites appeared in ‘drier’ environments. The actual wetness of the environment is not only determined by precipitation but also by evaporation. Wetness is defined as net precipitation, i.e. precipitation minus evaporation (P–E). Figure 4a shows the relationship of coals with P–E since the Late Paleozoic. Unlike the relationship with AMT and AMP, the relationship of coals with P–E does not show a major deflection at 250 Ma. Indeed, the relationship between the abundance of coals and P–E is remarkably constant. The median value of P–E was 300 mm yr^−1^ (Fig. 4b), and 50% of all coal records occurred within the P–E range of 100 mm yr^−1^–600 mm yr^−1^ (Fig. 4b). In general, most coal records were associated with positive P–E, and very few coal records appeared in regions of negative P–E.

Figure 4b shows the distribution of evaporites as a function of P–E. Again, the most striking feature is that the relationship between evaporite occurrence and P–E has remained nearly constant during the past 540 Myr. Evaporite records appeared mainly within the P–E range of 0 mm yr^−1^ to 300 mm yr^−1^, with the median value of 100 mm yr^−1^ (Fig. 4d). Evaporites were more scattered across latitude before 300 Ma. This scatter may be due, in part, to small sample sizes and the fact that the location of some of the continental terranes is less well known.

## VALIDATION AND PREDICTION

The above quantitative relationships of coals and evaporites for the deep time are consistent with that of peats and evaporites in modern climatic conditions [1,24], which showed that present-day peat-related AMT and AMP are higher than 10°C and between 500 mm yr^−^^1^ and 1000 mm yr^−1^, respectively, and that evaporite-related AMP ranges over 0–500 mm yr^−1^. Comparison of present-day peat and evaporite records with different climate simulations also shows that peak values of net precipitation for coals and evaporites are about 300 mm yr^−1^ and 100 mm yr^−1^, respectively [24].

**Figure 5.**
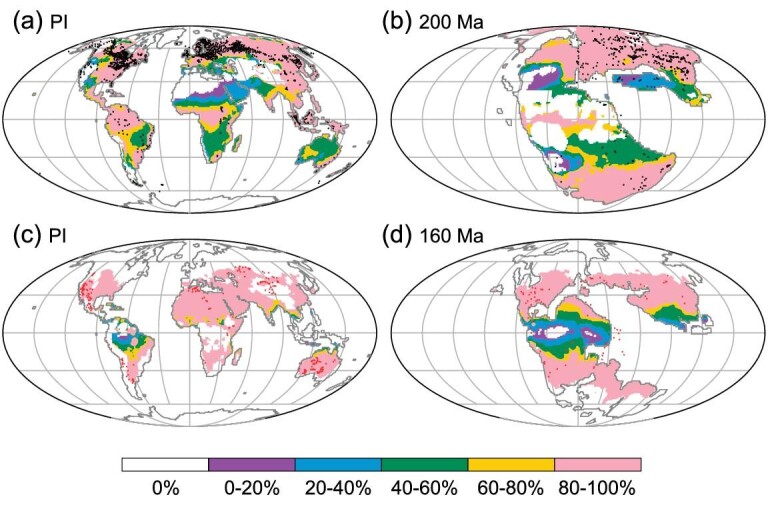
Comparison of locations of coals and evaporites with the predicted areas using simulated temperature and precipitation. Top panels: coals at PI (a) and 200 Ma (b). Bottom panels: evaporites at PI (c) and 160 Ma (d). Color bar denotes the percentage of coal and evaporite records that can be predicted with the temperature and precipitation criteria. Black dots in plots (a) and (b) denote coals, and red dots in plots (c) and (d) denote evaporites.

To further validate the reliability of our simulation results and the above quantitative relationships, we use simulated temperatures and precipitation to ‘predict’ the localities of coals and evaporates in the Phanerozoic, using the method in Ref. [1]. Two criteria are set for coals: monthly mean temperatures > 10°C and monthly mean precipitation > 40 mm. The percentage of those months with precipitation > 40 mm and temperatures > 10°C to the months with temperatures > 10°C is considered the possibility for coal prediction [1]. Considering the intolerance of plants to high temperatures, we set the prediction probability of coals to zero if all monthly mean temperatures > 30°C. Figure 5a shows the prediction map of peats, using the PI simulation results. Pink regions have the percentage above 80% (see online supplementary material for a colour version of this figure). About 83% peat records fall into such regions. Figure 5b shows the prediction map of coals in the very late Triassic (200 Ma). About 81% of coal records fall into these regions.

For evaporites, the percentage of prediction is defined as the ratio of those months with precipitation < 100 mm to those months with temperatures > 24°C. Figure 5c shows the prediction map of evaporites at the PI. Pink color denotes the regions with the percentage above 80%. About 76% of evaporites fall into such regions. Figure 5d shows the prediction map of evaporites in the late Jurassic (160 Ma). The number of eveporite records falling into such regions is 84%, with only small numbers of evaporite records that fall outside the regions, such as marine evaporites. These robust predictions suggest that the simulated temperatures and precipitation and their quantitative relationships with coals and evaporites are fairly reliable.

## DISCUSSION

By combining geological records of coals and evaporites with climate simulations, we have demonstrated that there are quantitative relationships of coals and evaporites with AMT and AMP, respectively. The relationship of coals is more complicated than that of evaporites because of the effects of plant evolution (pteridophytes vs. gymnosperms and angiosperms), the evolution of lignin-eating microbes (Agaricomycetes), and northward movement of landmasses [25,26]. These resulted in the dramatic shift of coal localities from the hot and humid tropics to the NH temperate and rainy belt at 250 Ma. We also show that all the Phanerozoic evaporite records nearly always appeared in the subtropical dry zones of both hemispheres.

It is notable that the relationships of net precipitation (P–E) with coals and evaporites remained nearly constant across time, with the median values of 300 mm yr^−1^ and 100 mm yr^−1^, respectively, despite radical changes in global climate during hothouse and icehouse intervals (Fig. 4). This indicates that the physical and chemical processes responsible for peat-coal and evaporite formations should be invariant with time. It is remarkable that this time-invariant relationship is robust, although there exist inherent uncertainties in continental positions of coals and evaporites and climate modelling. Moreover, the quantitative relationships are able to predict the locations of coals and evaporites in the past fairly well. The robustness suggests that our model of the evolving Earth System may accurately reflect past climate conditions. Of course, the quantitative relationships need to be examined in future simulation studies.

The results here also offer an example that can be used to derive quantitative relationships for other exogenetic ore deposits (e.g. bauxite) with surface temperature and precipitation. While it is well known that endogenic ore deposits (e.g. gold, copper, cobalt) are caused by tectonic and magmatic processes, the formation of exogenetic ore deposits are closely related to surface climate conditions prescribed by temperature and precipitation that control the relevant chemical and physical processes. The establishment of these quantitative relationships can also be used for deep-time climate reconstructions and predicting the locations of other exogenetic ore deposits.

## MATERIALS AND METHODS

### Data

The dataset of coals and evaporites used here is from the comprehensive compilation of coal and evaporite records [12] and the compilation of present-day peats and evaporates [6,27]. There are 5417 coal records (410 Ma–present) and there are 2329 evaporite records spanning the Phanerozoic. The number of coal and evaporite localities, plotted as a function of time, is shown in Supplementary Fig. 3. In this study, we have included only terrestrial evaporite deposits and some coastal evaporites. Marine evaporites have been excluded because their water source is not related to precipitation and their formation requires higher rates of evaporation than terrestrial evaporates [2].

The GPlates [28] and the global plate tectonic model [30] were used to calculate the paleo-latitude and paleo-longitude of the coal and evaporite localities. The reconstructed data were then plotted on the paleogeographic basemaps [29,30] (Supplementary Fig. 1). A total of 55 reconstructions were produced, one map for every 10 Myr interval during the Phanerozoic. Finally, the AMT, AMP, and P–E at each of the paleo-localities were extracted from the climate model results and tabulated for each time interval.

The observational precipitation data is from the Global Precipitation Climatology Project (GPCP) Version-2 Monthly Precipitation Analysis over 1979 to 2019 [13]. It merges precipitation estimates from low-orbit satellite microwave data, geosynchronous-orbit satellite infrared data, and surface rain gauge observations. The global and annual mean precipitation, averaged over 1979 to 2019, is 982 mm yr^−1^. Our simulated global and annual mean precipitation, averaged over the last 60 model years, is about 1062 mm yr^−1^ [10,11], which is slightly higher than the observation.

### Model and experiment design

Detailed information of the climate model used here and simulations setup can be found in references [10,11] and [31]. Here, we just present a brief introduction. Simulations were performed using the fully coupled atmospheric-oceanic Community Earth System Model version 1.2.2 [31] (CESM 1.2.2). The climate simulations were run by two steps. First, a lower-resolution version of atmospheric-oceanic coupled CESM 1.2.2 (3.75° × 3.75° in latitude and longitude) was used to run equilibrium simulations for each of the 55 paleogeographic reconstructions. In these simulations, reconstructed paleogeographies were prescribed by the digital elevation models [30]. The value of the solar constant was linearly increased at a rate of 0.08% per 10 Myr to the present value (1361 W m^−2^) [32]. Orbital parameters and atmospheric compositions, with the exception of CO_2_ were all set to present-day values.

Instead of using CO_2_ concentrations, we used reconstructed GMSTs [8] to constrain the level of CO_2_. For each simulation, CO_2_ concentration was increased or decreased until the simulated GMST matches the reconstructed GMST within ± 0.5°C at the equilibrium state. This ‘tuning’ often required multiple simulation runs. This procedure is described in more detail in references [10,11].

In the second step of simulations, monthly sea surface temperatures, sea ice, and annual mean vegetation derived from the lower-resolution simulations, averaged for the last 100 years, are used to drive the higher-resolution CESM atmosphere-land model (0.9375° × 1.25° in latitude and longitude). CO_2_ concentrations, paleogeography, and the solar constant remain the same as in lower-resolution simulations. All the higher-resolution simulations are integrated for 100 years to reach equilibrium states. AMT and AMP used here are the averages over the last 60 years.

## Supplementary Material

nwad051_Supplemental_Files
