# Supplementary material for: Quantifying climate conditions for the formation of coals and evaporites
Source: Natl Sci Rev. 2023 Feb 27;10(6):nwad051. doi: 10.1093/nsr/nwad051 (PMC10171626; doi:10.1093/nsr/nwad051)
Supplement: nwad051_Supplemental_Files [file nwad051_supplemental_files.zip › revision_SI_coals&evaps.docx]

Supporting Information for

**Quantifying Climate Conditions for the Formation of Coals and Evaporites**

Xiujuan Bao^1^, Yongyun Hu^1*^, Christopher R. Scotese^2^, Xiang Li^1^, Jiaqi Guo^1^, Jiawenjing Lan^1^, Qifan Lin^1^, Shuai Yuan^1^, Mengyu Wei^1^, Zhibo Li^1^, Kai Man^1^, Zihan Yin^1^, Jing Han^1^, Jian Zhang^1^, Qiang Wei^1^, Yonggang Liu^1^, Jun Yang^1^, Ji Nie^1^

^1^Laboratory for Climate and Ocean-Atmosphere Studies, Department of Atmospheric and Oceanic Sciences, School of Physics, Peking University, Beijing 100871, China.

^2^Department of Earth and Planetary Sciences, Northwestern University, Evanston, IL, 60208, USA.

**Corresponding author** Yongyun Hu, [yyhu@pku.edu.cn](mailto:yyhu@pku.edu.cn)

**Contents of this file**

Figures S1−S5

**Introduction**

This file provides the supplemental figures for the main manuscript.


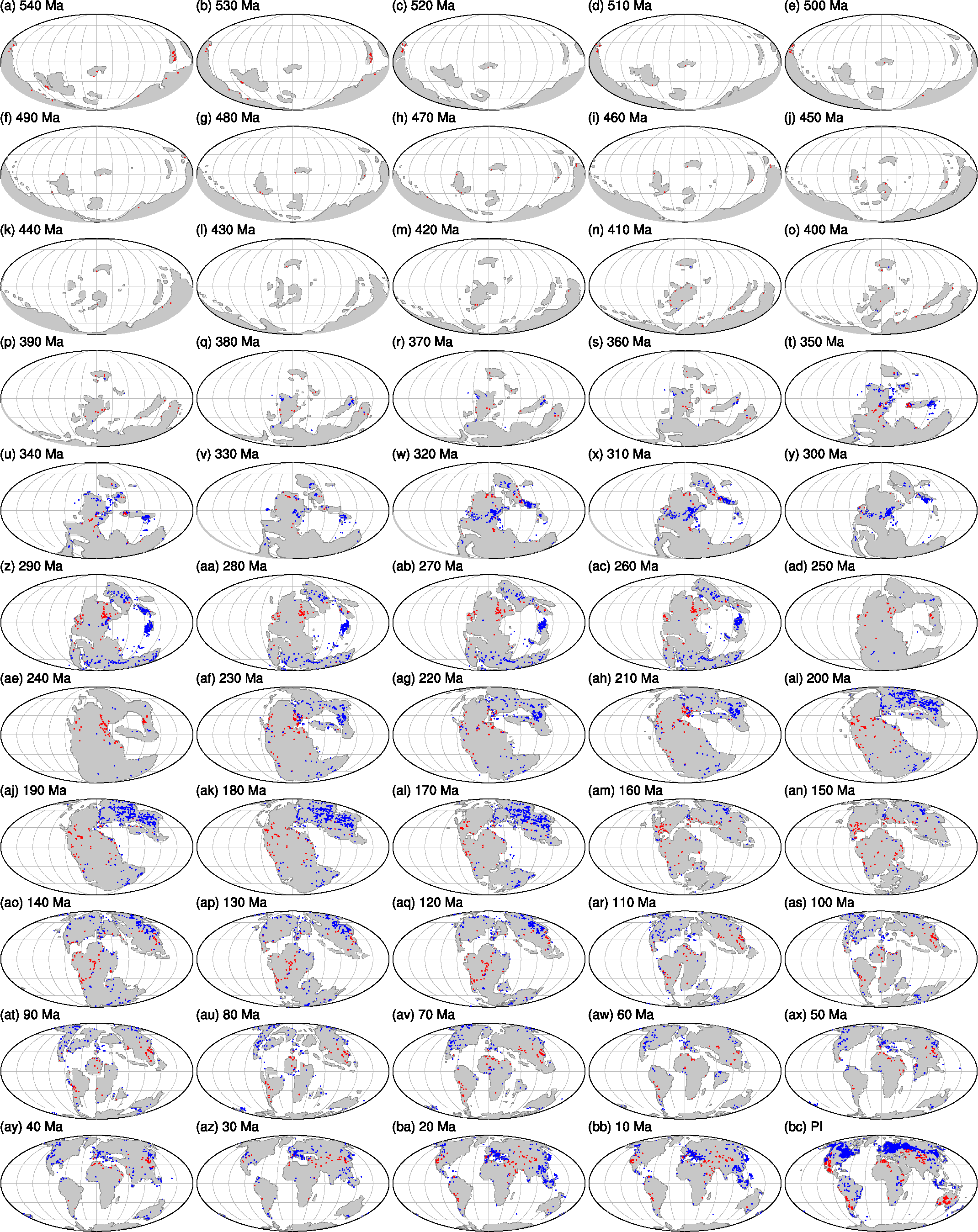


**Figure S1** Locations of coals and evaporites in the past 540 million years. Data is from Ziegler et al. (2003) [6] and Boucot et al. (2013) [13]. Blue dots: coals, red dots: evaporites.


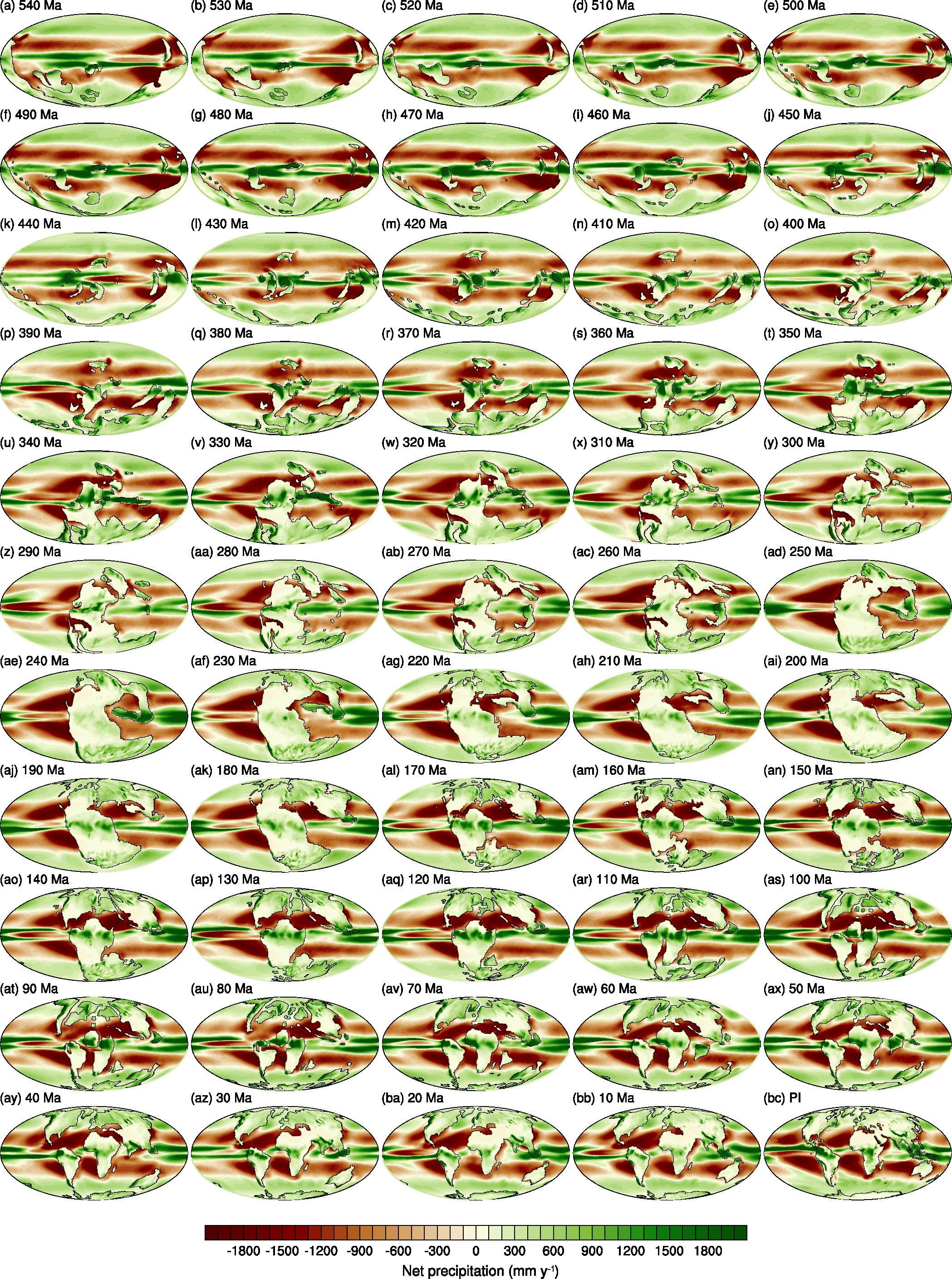


**Figure S2** Global distributions of simulated annual mean net precipitation in the past 540 million years.


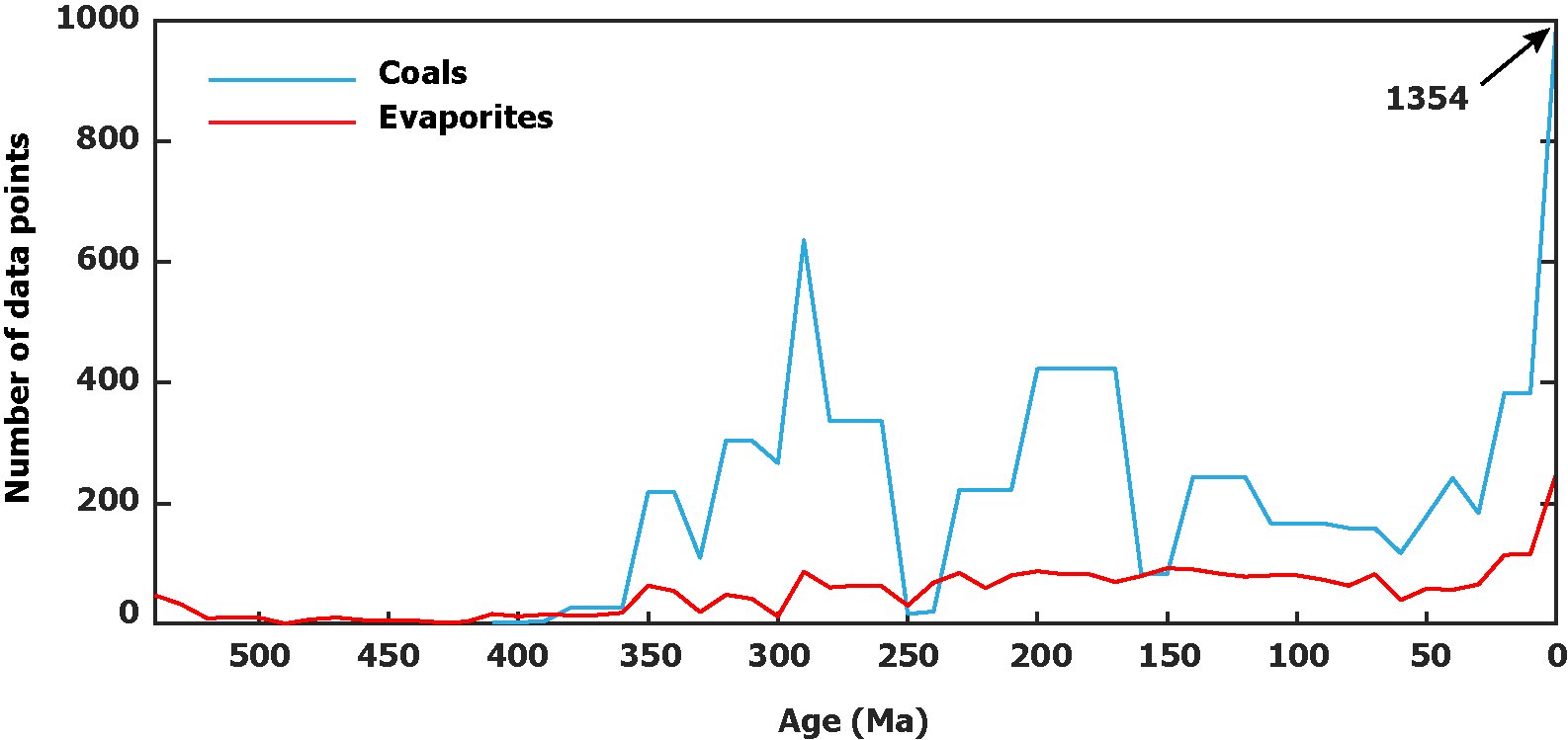


**Figure S3** Time series of numbers of coal and evaporite records in the past 540 million years. Note that the number of coal records at present is 1354, which is no completely displayed in the plot. Blue line: coals, and red line: evaporites.


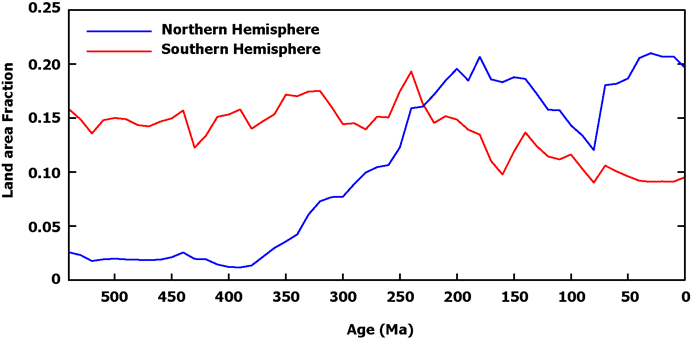


**Figure S4** Time series of hemispheric continental areas and subtropical continental areas in the past 540 million years. Blue line: Northern Hemisphere, and red line: Southern Hemisphere.


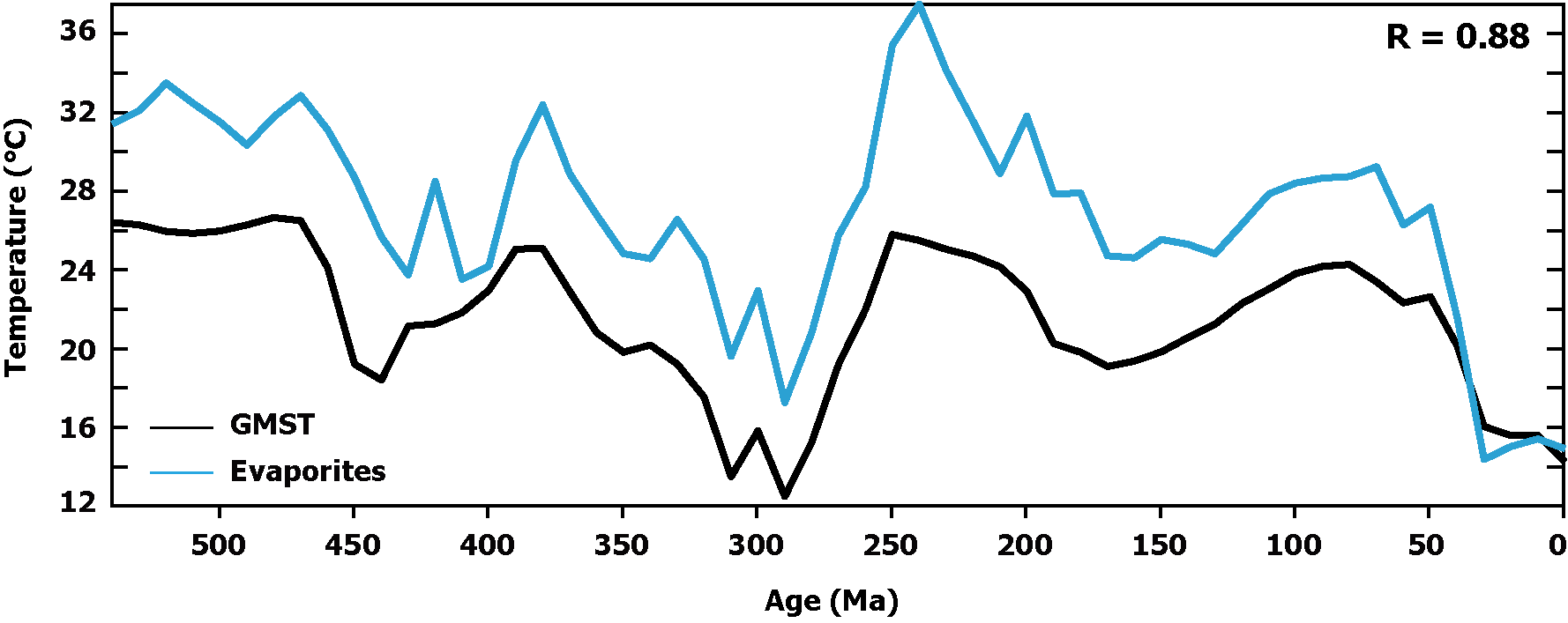


**Figure S5** Time series of annual and global mean surface temperatures and annual mean surface temperatures associated with evaporites. “R” is the correlation coefficient.
